# Study on Phylogenetic Relationships, Variability, and Correlated Mutations in M2 Proteins of Influenza Virus A

**DOI:** 10.1371/journal.pone.0022970

**Published:** 2011-08-02

**Authors:** Ly Le, Jacek Leluk

**Affiliations:** 1 School of Biotechnology, Ho Chi Minh International University, Ho Chi Minh City, Vietnam; 2 Department of Molecular Biology, Faculty of Biological Sciences, University of Zielona Góra, Zielona Góra, Poland; Georgia Institute of Technology, United States of America

## Abstract

M2 channel, an influenza virus transmembrane protein, serves as an important target for antiviral drug design. There are still discordances concerning the role of some residues involved in proton transfer as well as the mechanism of inhibition by commercial drugs. The viral M2 proteins show high conservativity; about 3/4 of the positions are occupied by one residue in over 95%. Nine M2 proteins from the H3N2 strain and possibly two proteins from H2N2 strains make a phylogenic cluster closely related to 2RLF. The variability range is limited to 4 residues/position with one exception. The 2RLF protein stands out by the presence of 2 serines at the positions 19 and 50, which are in most other M2 proteins occupied by cysteines. The study of correlated mutations shows that there are several positions with significant mutational correlation that have not been described so far as functionally important. That there are 5 more residues potentially involved in the M2 mechanism of action. The original software used in this work (Consensus Constructor, SSSSg, Corm, Talana) is freely accessible as stand-alone offline applications upon request to the authors. The other software used in this work is freely available online for noncommercial purposes at public services on bioinformatics such as ExPASy or NCBI. The study on mutational variability, evolutionary relationship, and correlated mutation presented in this paper is a potential way to explain more completely the role of significant factors in proton channel action and to clarify the inhibition mechanism by specific drugs.

## Introduction

The transmembrane matrix protein (M2) of the influenza virus plays a key role in the virus development in the host cell. It forms a pH-gated proton channel responsible for lowering pH of the intracellular environment of the virus [1]. This process acidifies the viral interior, which is required for the unpacking of the viral genome [2]. M2 protein also plays important role in the trans-Golgi network as the factor preventing premature structural rearrangement of the hemagglutinin during its transport to the cell surface of the host [3].

The specific inhibition of the viral M2 proton conductance action is a potential way to restrain the virus proliferation in the infected host cell. For that reason, detailed knowledge of the M2 structure-function relationship and its mutational variability are the subjects of investigation—to design effective, specific drugs against the particular strains of influenza virus [4]–[11]. Also a large-scale analysis on the evolution of the entire viral M gene in different hosts at both, genomic and protein level, is a rich source of information to achieve this goal [12]. The earlier studies of the M1 and M2 proteins [13] show that the influenza A viruses have evolved into at least four major host-related lineages. It has been observed that the M1 proteins indicate much slower evolutionary rate than M2 proteins, although M2 proteins of avian lineages are still relatively conservative [12]–[13]. Out of 42 analysed M proteins, 24.6% of M1 amino acid positions revealed some variability, while M2 proteins showed the divergence at 48.5% positions [13]. This difference in the evolutionary rate between M1 and M2 proteins may be a result of a greater response to host-immune selective pressure or structural constraints in case of M2 [12]–[13]. At present, 2 major antiviral drugs, Amantadine and Rimantadine, are being studied for their mechanism of action, specificity, and efficiency [4], [6], [8], [10], [11]. Although the experimental structure data of viral M2 protein are available [4], [10], the models of the interaction of the Amantadine/Rimantadine with the proton channel protein are incompatible. According to some authors, Rimantadine binds at 4 equivalent sites near the gate on the lipid-facing side of the channel and stabilizes the closed conformation of the pore [10].

According to other reports, only one molecule of the drug (Amantadine) binds to the helical bundle of the transmembrane part of M2 and is located at the N-terminal side in the lumen of the pore [4]; thus, it forces the proton channel gate conformation to its functionally closed form.

Additionally, the involvement of particular residues in the mechanism of the proton conductance inhibition by these drugs is not clear [6]–[8], [10], [11], [14].

The aim of this work is to give possible answers to some of the questions about the significant residues of M2 for its action and for the residues that possibly interact with the drug, hindering the proton transport through the membrane; additionally this work studies the mutational variability within the viral M2 proteins. The identification and characterization of the correlated mutations occurring within the M2 molecule can serve as additional sources of information about functionally significant residues/regions of the viral proton channel protein.

In our work, we focused on the matrix protein 2 from Influenza A virus [10] (A/Udorn/307/1972(H3N2), pdb code 2RLF) and completed a comparative study with 92 homologous viral M2 proteins.

## Results and Discussion

### Multiple Sequence Alignment

**Figure 1:**
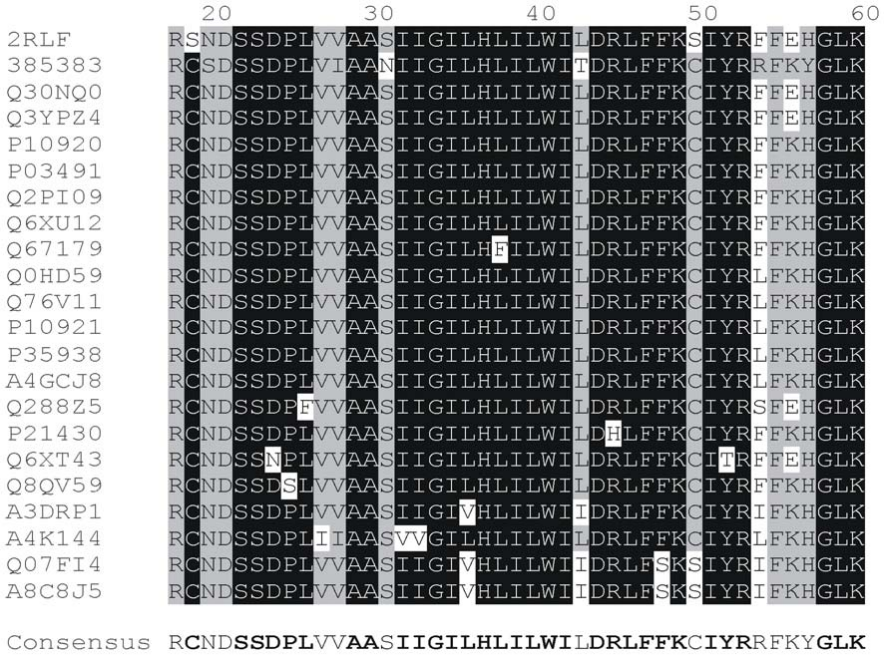
Multiple sequence alignment (part) of the influenza virus M2 proton channel proteins. The consensus sequence for aligned 43 amino acid fragments was obtained for the parameters: identity 96.65% (88), significance 29.35% (27), gaps 50% (46).

The 92 M2-like sequences revealing significant identity/similarity to the 2RLF M2 proton channel of influenza A virus (A/Udorn/307/1972(H3N2), PDB code 2RLF, MMDB ID: 62125, GI:166235427) were selected with protein BLAST application (blastp) at the default parameters of search [15]–[17]. The multiple alignment of the selected sequences was achieved with the aid of ClustalX [18]–[19] and verified with the algorithm of genetic semihomology [20]–[23]. As for the virus proteins, the sequences show very high conservativity (Fig. 1). About 3/4 positions of the 92 aligned 43 amino acid fragments that correspond to the protein 2RLF are occupied by one residue in over 95% of sequences. This observation is consistent with the other results [12]–[13] concerning the study of evolution of the entire viral M gene. However the M2 gene reveals much higher evolutionary (higher mutational variability) rate than M1 [12]–[13]. There were observed some variable positions that significantly differentiated 2RLF from other family members. The most unique differences concern serines at positions 19 and 50 of protein 2RLF, whereas in most related sequences those positions are occupied by cysteines. Serine at the position 2 occurs only in protein 2RLF, while the Ser33 was found in 3 other sequences. Also the tetrapeptide close to C-terminus (FFEH in 2RLF) is sufficiently variable as a potential site of indiviual specificity that may distinguish protein 2RLF properties from other family members. These variable sites should be the starting point of interest for molecular design of the ligands specifically affecting only the activity of M2 proton channel of influenza A virus.

The verified correct multiple alignment was used to construct the consensus sequence for complete length of M2 proteins and for the 43 amino acid fragment corresponding to 2RLF (Fig. 1). The consensus sequence was constructed with the aid of Consensus Constructor [24] at the threshold parameters of identity = 96.65%, significance = 29.35%, and gaps = 50%. It describes the significant features of M2 family and serves as a query sequence for effective search of the evolutionary and functionally related proteins.

### Phylogenetic analysis of M2 family

**Figure 2:**
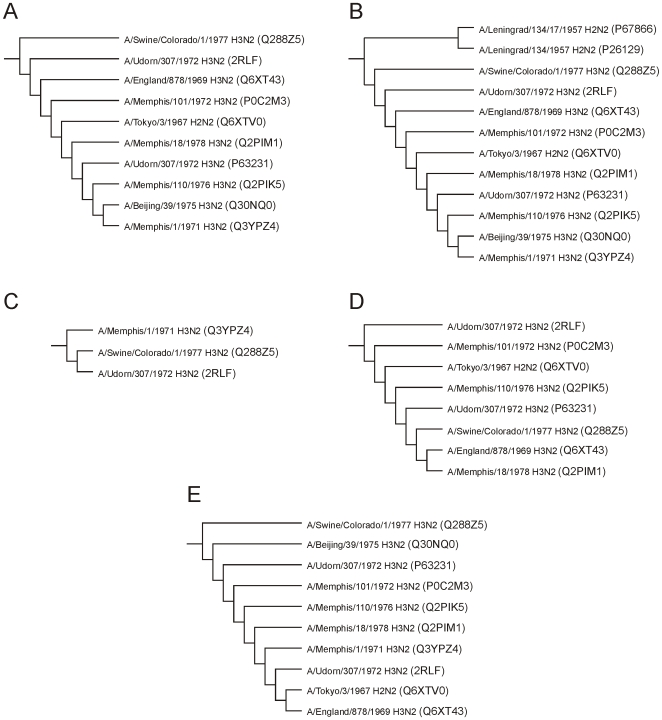
The cladograms of 2RLF cluster obtained with the different approaches. The results were obtained with the aid of the following software: (A) ClustalX, (B) SSSSg, (C) Phylip (maximum parsimony), (D) Phylip (maximum likelihood), (E) ConSurf.

The phylogenetic trees were constructed by 5 independent methods with the aid of ClustalX [18]–[19], SSSSg [25], ConSurf [26]–[28] and Phylip [29]. Despite the very high identity score between the aligned sequences, each approach yielded a different result with respect to the tree topology and the branch distance lengths. This demonstrates that for better reliability, the phylogenetic analysis should not be limited to one method only; rather, results need to be verified using several independent approaches. The individual trees constructed by different methods were compared and analyzed in terms of the occurrence of similar clads and clusters. The phylogenetic trees were constructed through each approach in several versions (cladograms, phylograms, and unrooted trees) and algorithms (maximum parsimony, maximum likelihood, and SSSSg identity/length/distribution analysis). There were 2 sets of input multiple alignments used: the complete sequence alignments and the alignments of 43 amino acid fragments corresponding to the protein 2RLF. Both approaches gave similar results in general, although the results for 43 amino acid fragments were more univocal. In the latter case, all 5 methods selected the group of 10 sequences closely related to the protein 2RLF (Fig. 2). As expected, the 9 sequences revealing very close evolutionary relationship to the protein 2RLF are the viral M2 proton transport proteins from H3N2 strains (UniProt accession numbers: Q288Z5 (A/Swine/Colorado/1/1977 H3N2), Q6XT43 (A/England/878/1969 H3N2), P0C2M3 (A/Memphis/101/1972 H3N2), Q6XTV0 (A/Tokyo/3/1967 H2N2), Q2PIM1 (A/Memphis/18/1978 H3N2), P63231 (A/Udorn/307/1972 H3N2), Q2PIK5 (A/Memphis/110/1976 H3N2), Q30NQ0 (A/Beijing/39/1975 H3N2) and Q3YPZ4 (A/Memphis/1/1971 H3N2).

The comparative phylogenetic analysis of the complete protein sequences were not as clear as for the 43 amino acid fragments corresponding to the protein 2RLF. However they identified the cluster of 7 closely related proteins out of 9 listed by the above result. In addition, there were also selected 2 other proteins from strain H2N2 indicating the potential close relationship to this group (UniProt accession numbers: P21430 (A/Ann Arbor/6/1960 H2N2) and Q67179 (A/Korea/426/1968 H2N2)).

### Mutational Variability of M2 Proteins

**Figure 3:**
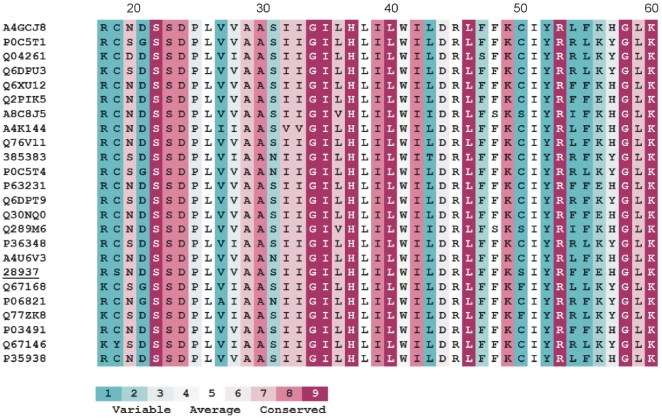
ConSurf color-coded multiple sequence alignment of M2 family. Only a part of the multiple alignment is shown. The different colors indicate the variability range of each position. See the text for details.

The multiple alignment (partial) of M2 proteins with color scale of variability/conservativity (ConSurf) is shown in Fig. 3.

**Figure 4:**
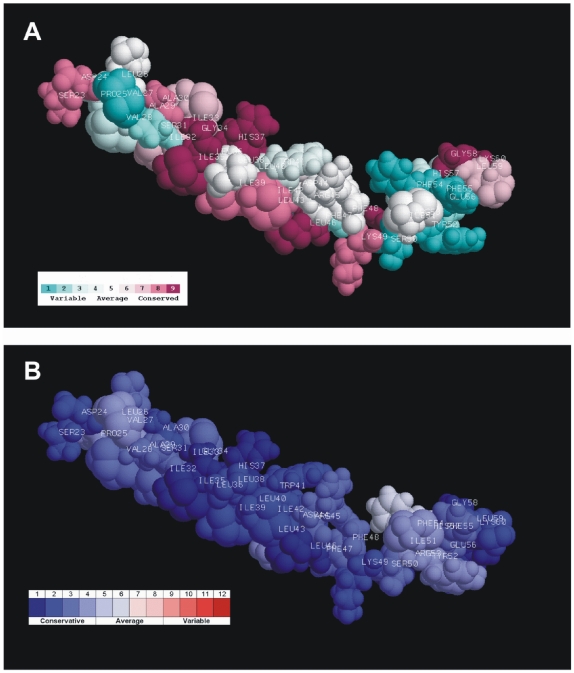
The mutational variability color scale plotted on the chain A of 2RLF protein structure. The results obtained with the aid of ConSurf (A) and Talana (B).

Because the viral M2 proteins are considerably conservative (Fig. 1), the mutational variability analysis concerned only some selected positions/fragments. The mutational variability was investigated and visualized with the aid of ConSurf [26]–[28] and Talana [30]. The results obtained by both applications were consistent with each other (Fig. 4).

The mutational variability was analyzed for M2 43 amino acid fragments corresponding to 2RLF. Nine positions are occupied by one residue, in about 27 positions occur 2 or 3 different residues, 6 positions reveal a variety of 4 residues, and 1 position admits 6 different residues. This most variable position is position 54, and in 2RLF it is occupied by phenylalanine. It reveals mutational correlation with other positions of lower variability (see chapter “Search for the Correlated Mutations”). It was expected that the residues significant for the M2 function should occupy mostly conservative positions. However they are within the variety range from 1 to 4. The average variability for 15 functional positions of the fragment corresponding to 2RLF (2.33 residues/position) is also at the level of the total variability of this fragment (2.49 residues/position).

### Search for the Correlated Mutations

Since the viral M2 proteins are conservative, there were not expected many intramolecular mutational correlations. However, some clusters of significant mutational correlation were found with the aid of the Corm program [31]. The clusters of identified mutational correlation are listed on Table 1.

**Table 1:** Correlated mutations within the M2 fragments corresponding to the protein 2RLF.

Reference position (amino acid)	Sequence number	Correlated positions (amino acids)
27 (I)	6	31 (S)
27 (T)	3	31 (KN)
27 (V)	82	31 (NS)
36 (L)	88	43 (FLT), 54 (CFLRS)
36 (V)	4	43 (I), 54 (I)
43 (F)	3	28 (AI), 36 (L), 54 (R)
43 (I)	4	28 (V), 36 (V), 54 (I)
43 (L)	84	28 (AIV), 36 (L), 54 (CFLRS)
50 (C)	84	28 (AIV), 54 (CFILRS), 57 (HY)
50 (F)	4	28 (I), 54 (LR), 57 (Y)
50 (S)	4	28 (V), 54 (FI), 57 (H)
54 (F)	18	36 (L), 43 (L)
54 (I)	4	36 (V), 43 (I)
54 (L)	11	36 (L), 43 (L)
54 (R)	57	36 (L), 43 (FLT)

Corm results for the parameters of minimum counts of amino acids: 3 and maximum identity threshold: 97%.

**Figure 5:**
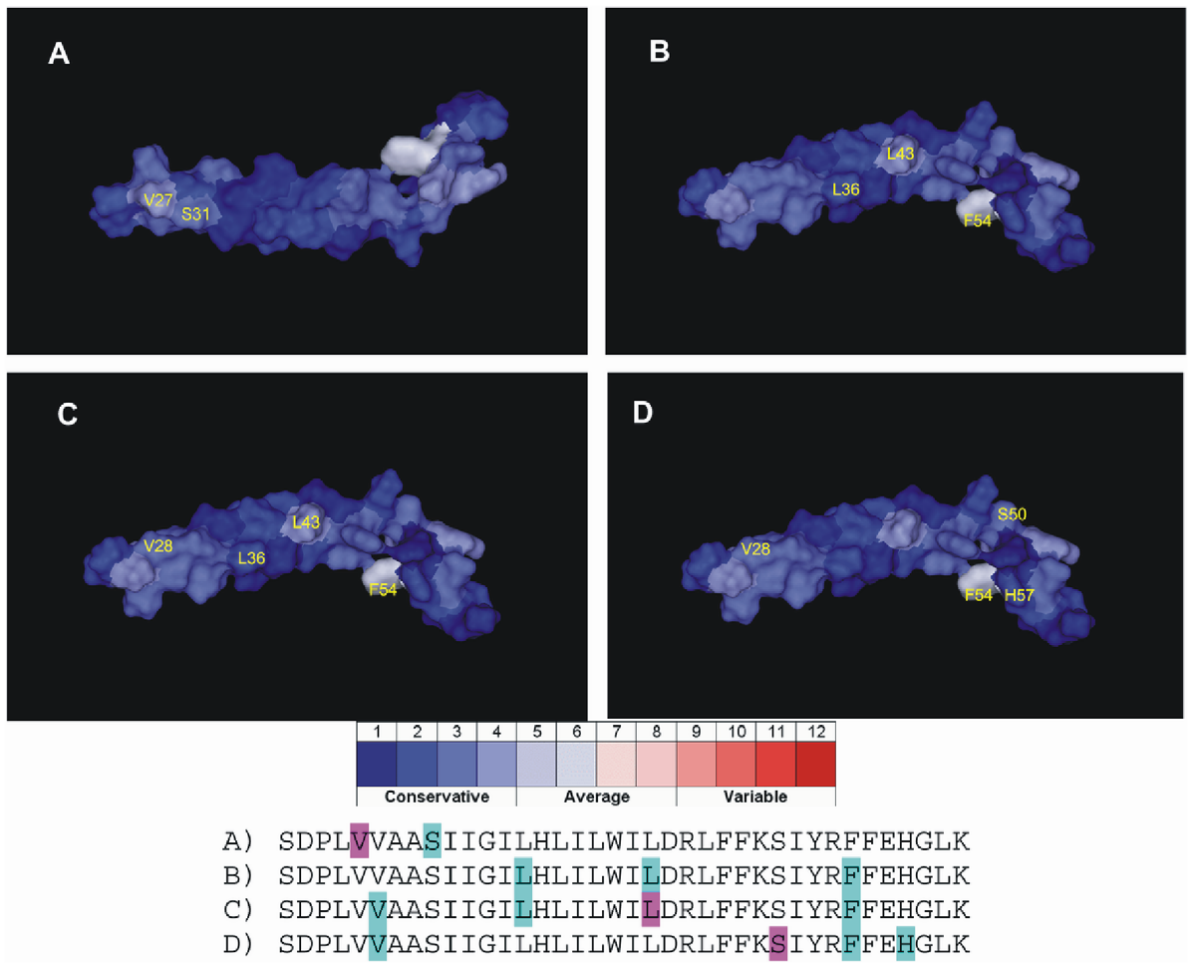
Mutationally correlated positions plotted on 2RLF chain A presented in the mutational variability mode. The variability scale is shown below the structures. The sequences indicate the location of positions (cyan) correlated to the reference position (purple) for the structures A, B, C, and D respectively. In B, the reference position is not indicated due to the mutual correlation for all 3 positions (using each of those positions as the reference gives the same cluster of correlation).

The positions revealing the correlation have been plotted on the 2RLF structure to show their locations and mutual distances in the molecule (Fig. 5).

The most explicit cluster of correlated mutations is observed for the positions 36, 43, and 54, which in 2RLF are occupied by L36, L43, and F54 (Fig. 5B). In the previous reports, only L43 is indicated as involved directly in proton transfer action, as it is the residue of one of the possible binding sites of the specific proton transfer inhibitors (Amantadine and Rimantadine). The important role of that residue is also caused by its adjoining location to the D44, which is the functional proton channel lock of M2 protein [6], [10]. However, 2 other correlated positions of the cluster are not mentioned as positions that may play an important role in either the proton transfer action or in the inhibitor binding. The cluster L36-L43-F54 is the only set that reveals the mutual correlation in all directions (i.e., taking any of these positions as a reference position for mutational correlation search reveals 2 other positions as correlated). Only in the case of position 43 as the reference, one more positions (28 occupied by Val in 2RLF) is identified as correlated (Fig. 5C). Two other correlation clusters concerned the positions 27–31 (V27 and S31 in 2RLF) and 28-50-54-57 (V28, S50, F54, and H57 in 2RLF) respectively (Figs. 5A and D). The correlated pair 27–31 is especially interesting as those positions play an important role in proton channel function [4], [5], [7], [8], [10], [11], and mutations at these positions increase the resistance to Amantadine/Rimantadine [4], [7], [8], [11]. The cluster 28-50-54-57 consists of the positions that have not been reported as directly involved in the proton transport apparatus of M2. These results suggest that some positions crucial for the protein functioning have not been identified or described. The analysis of mutational correlation can serve as an additional source of information to explain the complete mechanism of action of the viral M2 proteins.

The most rigorous correlation is observed for mutual dependence of polar amino acid occurrence. For example valine at the position 14 admits the occurrence of only isoleucine at positions 21 and 32 (Tab. 1). Also both Ile21 and Ile32 accept only Val14.

An interesting correlation has been observed for the pair 27–31 (Tab. 1). Both positions play an important role in the proton channel action. In most M2 proteins, position 31 is occupied by serine, which is identified as a pore-lining residue opening the pore at the His37 gate^7^. According to other reports [10], Ser31 is a significant helix-helix packing interface factor. Val27 is also known as a pore-lining residue [9], [10]. Moreover, it has been described as the secondary gate of M2 proton channel [11]. Val27 is located at the narrowest point of the N-terminal region of the pore, and it interacts with proton channel inhibitors such as Amantadine and Rimantadine [4]. Ser31 does not interact directly with the drug [8] although mutation at the position 31 results in resistance to Amantadine [4], [8], [11]. The mutational correlation within the pair 27–31 suggests cooperation of the primary and secondary gate of proton channel. The variety range of the correlated positions within a cluster is diverse. Within one cluster may occur positions of residue variety from 2 to 6.

The most often occurring position in mutationally correlated clusters is position 54 (Phe in 2RLF). It is present in 3 clusters shown on Fig. 5. This position is also the most variable position in analyzed M2 proteins. These results indicate the strong positive selective pressure on that site and its potentially significant role in determining the M2 specificity of each strain.

In summary, it is established that the positions 27, 28, 31, 36, 43, 50, 54, and 57 are involved in mutational correlation clusters, then they play a potential role in the proton channel activity of M2. Only some of these positions have been described previously as significant agents of the proton transfer mechanism. The role of positions 28, 36, 50, 54, and 57 is unknown. However the data concerning position 57 are consistent with the results of Furuse et. al. [12] who indicated that this position revealed significant positive selection and that the consensus amino acid between human and avian influenza was different. The analysis of their participation in the proton transfer mechanism or interaction with channel inhibitors should provide more data concerning the Amantadine and Rimantadine inhibition mechanism, which is still not clear.

### Outlook on rational drug design

Current antiviral drugs, Amantadine and Rimantadine, targeting the M2 channel have now become ineffective due to key mutations that alter the structure of the M2 channel. In 2005, Amantadine-resistant strains of influenza became nearly ubiquitous (99% of circulating strains were resistant), and consequently, the only drugs available for severe flu infections were the scarcer and costlier neuraminidase inhibitors, which target a different mechanism in flu replication. Even more alarmingly, the recent increase in oseltamivir resistance of both the avian H5N1 and H1N1pdm strains makes it crucial to develop newly effective M2 channel blockers [32]. Recent studies have reported that the combination therapy of the M2 inhibitors and neuraminidase inhibitors might be an effective means of reducing morbidity and mortality in treatment of flu [33]. Mutations inducing M2 inhibitor drug resistance, which are known so far, include S31N, L26F, V27A, A30T, G34E, L38F [34]. From our study, we suggest focusing also on the mutations at positions 28, 36, 50, 54, and 57 for rational design of new antiviral drugs that are expected to have broader effect on different variants of influenza.

## Materials and Methods

### The databases, multiple sequence alignment an consensus sequence

The template protein used in the study was the matrix protein 2 from Influenza A virus (A/Udorn/307/1972(H3N2)) [10]. The amino acid sequence and structure of this protein was obtained from Protein Data Bank (http://www.rcsb.org/pdb) (pdb code 2RLF).

The virus M2 protein sequences were taken from the UniProtKB/Swiss-Prot protein sequence database (http://www.expasy.org/sprot/). The M2 sequences revealing significant identity/similarity scores to the 2RLF were selected by protein BLAST (blastp) [15]–[17] at the default values of search.

For the preliminary multiple sequence alignment ClustalX [18]–[19] was used, then the alignment was verified and corrected if necessary by manual analysis following the algorithm of genetic semihomology [20]–[23]. The verification concerned the possible genetic relationship between compared positions which were non-identical (possible replacements by single transition/transversion). The consensus sequence of the aligned M2 proteins was constructed with the aid of Consensus Constructor [24].

### Phylogenetic trees

The phylogenetic trees were constructed on the basis of different algorithms of distance calculation and with the aid of different applications. The programs used for cladogram and/or phylogram construction were: ClustalX [18]–[19], SSSSg [25] (freely accessible at: http://atama.wnb.uz.zgora.pl/~jleluk/software/wlasne/ssssg/ssssg.zip), ConSurf [26]–[28] and Phylip [29]. The phylogenetic trees were constructed on the basis of the aligned complete sequences and of the 43 residue fragment corresponding to 2RLF.

### Study on evolutionary variability

The mutational variability/conservativity of the aligned homologous positions and regions was analysed with the use of ConSurf [26]–[28] and Talana [30] (available at: http://www.bioware.republika.pl/). The variability pattern plotted on the 2RLF structure was visualised by Rastop2.2 (http://www.geneinfinity.org/rastop).

### Correlated mutations

The correlated mutations were identified, localized and analysed with the aid of Corm [31]. The software is freely available at http://atama.wnb.uz.zgora.pl/~jleluk/software/wlasne/corm.jar. The identified clusters of correlated positions were visualised on the 2RLF template structure by DSVisualizer1.7 of Accelrys (http://accelrys.com/products/discoverystudio/visualization-download.php).

### The availibility of original software generated by authors and co-workers

The original applications such as Consensus Constructor, Talana, Corm, and SSSSg are freely available at the addresses described above. Also they are available directly upon request sent to the authors. Additionally the authors are willing to assist in appropriate effective running all these application in case of any problems.
